# Comparative transcriptomics in B73-teosinte near-isogenic maize lines reveals key defense signaling and phytoalexins in response to *Cercospora zeina* infection

**DOI:** 10.3389/fpls.2025.1580016

**Published:** 2025-06-05

**Authors:** Xiaomeng Sun, Ruiyu Zhang, Zongping Wang, Haotian Zhang, Hairun Wen, Junli Zhang, Naibin Yu, Zhe Wang, Huanhuan Tai, Qin Yang

**Affiliations:** ^1^ State Key Laboratory of Crop Stress Resistance and High-Efficiency Production, College of Agronomy, Northwest A&F University, Yangling, China; ^2^ Hainan Research Institute of Northwest A&F University, Sanya, China; ^3^ State Key Laboratory of Crop Stress Adaptation and Improvement, Henan Joint International Laboratory for Crop Multi-Omics Research, School of Life Sciences, Henan University, Kaifeng, China

**Keywords:** maize, gray leaf spot, transcriptome, terpenoid metabolism, jasmonic acid

## Abstract

Gray leaf spot (GLS), caused by *Cercospora zeae-maydis* and *Cercospora zeina*, is a devastating foliar disease affecting maize production worldwide. However, the defense mechanisms underlying GLS resistance are poorly understood. A major quantitative trait locus (QTL), *Qgls8*, associated with GLS resistance, was previously identified on maize chromosome 8 bin 8.06. Here we conducted transcriptome analyses on leaves from a pair of B73-teosinte near-isogenic lines (NILs) with contrasting *Qgls8* alleles infected with *C. zeina* at 0, 4, 8, and 12 hours post inoculation (hpi). A total of 1225 up-regulated genes (URGs) were identified in the resistant line Qgls8-R compared with the susceptible line Qgls8-S across the four time points. By contrast, 908 URGs were identified in Qgls8-S. The URGs in Qgls8-R were significantly enriched in metabolic processes, phytohormone signaling, and response to biotic stress, while the URGs in Qgls8-S were mostly involved in plant growth and developmental processes. Additionally, *C. zeina*-induced URGs were consistently enriched in terpenoid metabolism and jasmonic acid (JA) signaling. Terpene- and JA-related genes showed increased expression at least at one time point after *C. zeina* infection which were confirmed by RT-qPCR. Furthermore, metabolite quantification indicated higher levels of JA and its isoleucine conjugate (JA-Ile) in Qgls8-R compared to Qgls8-S. Weighted gene co-expression network analysis (WGCNA) identified the module “turquoise”, which exhibited the highest positive correlation with Qgls8-R and was related to JA signaling. These findings suggest that the defense response mediated by terpenoid metabolism and the JA signaling pathway plays crucial roles in enhancing GLS resistance following *C. zeina* infection.

## 1 Introduction

Plants often initiate their innate immune systems to respond to pathogen attacks, including pattern-triggered immunity (PTI) and effector-triggered immunity (ETI) (Jones and Dangl, 2006; Couto and Zipfel, 2016; Yuan et al., 2021; Ngou et al., 2022a, 2022). PTI is triggered by the perception of pathogen-associated molecular patterns (PAMPs) or damage-associated molecular patterns (DAMPs) via pattern recognition receptors (PRRs) on the surface of plant cells. ETI is triggered by the recognition of effectors via intracellular nucleotide-binding domain, leucine-rich-repeat containing receptors (NLRs), thereby leading to hypersensitive response (HR) (Tsuda and Katagiri, 2010; Cui et al., 2015; Couto and Zipfel, 2016; Peng et al., 2018). Plant responses to pathogens involve the production of phytohormones and phytoalexins (Tsuda and Katagiri, 2010; Yuan et al., 2021; Zhang and Dong, 2022). Upon the perception of pathogen infection, the phytohormone jasmonic acid (JA) conjugates with isoleucine to activate jasmonoyl-isoleucine (JA-Ile)-triggered signal transduction (Chini et al., 2007; Thines et al., 2007; Fonseca et al., 2009; Sheard et al., 2010; Zhang et al., 2015). In maize, the major phytoalexins belong to terpenoids, including sesquiterpenes such as zealexins and α/β-costic acids, as well as diterpenes such as kauralexins and dolabralexins, which usually restrict the growth of pathogens (Huffaker et al., 2011; Ding et al., 2017; Mafu et al., 2018; Ding et al., 2019).

Maize (*Zea mays* L.) is one of the key staple crops for feeding the growing global population. Gray leaf spot (GLS), one of the most devastating foliar diseases in maize worldwide, is caused by the necrotrophic fungi *Cercospora zeae-maydis* and *Cercospora zeina*. Since its first discovery in Alexander County, Illinois, USA, GLS has been prevalent in the United States, South Africa, Brazil and China due to the expansion of the causal fungal pathogens, resulting in a yield loss of up to 60% (Ward et al., 1997, 1999; Dunkle and Levy, 2000; Liu and Xu, 2013; Nsibo et al., 2019). GLS resistance is predominantly inherited as a quantitative trait, and a number of quantitative trait loci (QTLs) have been identified on all the 10 maize chromosomes (Zhang et al., 2012; Berger et al., 2014; Xu et al., 2014; Benson et al., 2015; Zhang et al., 2017; Du et al., 2020; Lv et al., 2020). Among them, only five genes have been cloned, including *ZmWAKL* (Cell-wall-associated receptor kinase-like protein gene) (Zhong et al., 2024) and *ZmWAK02* (RD wall-associated kinase gene) (Dai et al., 2024), as well as three multiple disease resistance genes, *ZmCCoAOMT2* (Caffeoyl-CoA *O*-methyltransferase gene (Yang et al., 2017), *ZmMM1* (*Mexicana lesion mimic 1*) (Wang et al., 2021), and *ZmCPK39* (Calcium-dependent protein kinase gene) (Zhu et al., 2024).

Teosinte, as the ancestor of modern maize, possesses potential rare novel alleles for maize improvement (Piperno et al., 2009; Kistler et al., 2018; Yang N. et al., 2023), such as *UPA2* (*Upright Plant Architecture2*) for leaf angle (Tian et al., 2019), and *THP9* (*TEOSINTE HIGH PROTEIN 9*) for seed protein content (Huang et al., 2022). In maize disease resistance, the teosinte-derived *ZmMM1* allele confers enhanced resistance to northern leaf blight, GLS, and southern corn rust (Wang et al., 2021). Six QTLs for GLS resistance have been identified using a population of 693 teosinte introgressed near-isogenic lines (NILs) in maize inbred line B73 background, which are located in bins 2.04, 3.06, 4.07, 5.03, 8.06, and 9.03 (Lennon et al., 2016; Liu et al., 2016). The QTL in bin 8.06 has been fine-mapped to an approximately 130-kb interval, which were designated as *Qgls8* (Zhang et al., 2017). A pair of B73-teosinte NILs, hereafter named as Qgls8-R/Qgls8-S, carrying different teosinte accession introgressions in the *Qgls8* region, exhibited a significant difference for GLS resistance (Lennon et al., 2016; Zhang et al., 2017).

This study aimed to elucidate the molecular mechanisms underlying GLS resistance in maize. We conducted a comprehensive transcriptome analysis using the two NILs Qgls8-R and Qgls8-S, which were inoculated with the fungal pathogen *C. zeina*. The comparative transcriptome analysis was performed at four time points after *C. zeina* infection to identify up-regulated genes (URGs) specific to each line. Functional analysis of these URGs revealed several critical pathways involved in defense signaling against *C. zeina*. The activation of these signaling pathways was confirmed by quantitative analyses of representative genes expression and metabolites accumulation levels during pathogen infection. Furthermore, weighted gene co-expression network analysis (WGCNA) was performed across 24 samples. These findings contribute to a deeper understanding of the molecular mechanisms underlying GLS resistance.

## 2 Materials and methods

### 2.1 Plant materials

*Qgls8*, a QTL for GLS resistance located in maize bin 8.06, has been previously reported (Zhang et al., 2017). A pair of NILs was developed by introgressing teosinte accessions into the B73 background, which carry different teosinte alleles at *Qgls8* region, termed as Qgls8-R and Qgls8-S (Lennon et al., 2016; Liu et al., 2016). Qgls8-R and Qgls8-S were provided by Dr. Peter Balint-Kurti at the Department of Entomology and Plant Pathology of North Carolina State University. Qgls8-R exhibited increased GLS resistance, while Qgls8-S exhibited decreased GLS resistance. Transcriptome sequencing was carried out on both Qgls8-R and Qgls8-S. The genomic DNA was extracted from both Qgls8-R and Qgls8-S. The Maize6H-60K SNP array was used to detect the genetic background of the two NILs.

### 2.2 GLS resistance evaluation in field trials

In the summers of 2023 and 2024, Qgls8-R and Qgls8-S were planted in Tongchuan, Shaanxi Province of China, where GLS occurs naturally each year. The field experiment was set up with two replicates of each line in both years. In all trials, each replicate was made up of three rows. Fifteen seeds were sown in a single row which was 3 m in length and spaced 0.6 m apart. Disease scoring was performed three times after anthesis. The interval between each disease scoring was approximately 10 days. The GLS symptoms were scored on a scale of 1 to 9, where “1” for the most susceptible score indicated that everything was completely dead; 2 indicated that almost all of the plant tissues had withered; 3 indicated that the leaf above the ear was withered; 4 indicated that spots of the leaf above the ear had merged together; 5 indicated that spots on the ear leaf had merged together; 6 indicated that many spots appeared on the ear leaf and was separate from each other; 7 indicated that a few spots appeared on the ear leaf; 8 indicated that a few spots appeared on the leaf below the ear; and “9” for the most resistant score indicated that plants showed no disease symptom (Lennon et al., 2016; Sermons and Balint-Kurti, 2018).

### 2.3 Artificial inoculation and sample collection

The two NILs, Qgls8-R and Qgls8-S, were grown in greenhouse at 25 °C under 14 h light/10 h dark conditions. *C. zeina* was isolated from maize leaves with GLS symptoms in the fields of Baoji, Shaanxi Province, China. The pathogenic fungi were cultured for ten days on MLPCA medium (20 g/L maize leaf powder + 2 g/L CaCO_3_ + 15 g/L agar) in the dark at 25 °C. The spores of *C. zeina* were collected using the elution solution (0.5 g/L agar + 0.5 mL/L Tween 20; autoclaved at 121°C). The spore suspension was diluted to 5×10^4^ conidia/mL and sprayed on both sides of the fourth leaf of inoculated plants at the four-leaf stage. Subsequently, the inoculated plants were humidified for 24 hours using humidifier. *C. zeina*-inoculated leaves were sampled at 0, 4, 8, and 12 hours post inoculation (hpi) for transcriptome sequencing.

### 2.4 Transcriptome sequencing and analysis

The fourth leaves of maize plants inoculated with *C. zeina* were sampled at 0, 4, 8, and 12 hpi. Three biological replicates were set for each time point, and each biological replicate was derived from three independent plants. All samples were sequenced based on Illumina X plus platform using a 150 bp pair-end sequencing strategy in Biomarker Technologies. Clean reads were mapped to B73_v4 reference genome (Zm-B73-reference-4.0 genome. fasta) using STAR 2.7.10a. The number of counts per gene was obtained using StringTie. Gene expression was normalized to transcripts per million (TPM). DEseq2 was used to screen URGs with a significance threshold of Fold Change > 1.5 and padj ≤ 0.05 (Lecourieux et al., 2020). URGs were identified in Qgls8-R compared with Qgls8-S or in Qgls8-S compared with Qgls8-R. URGs responding to *C. zeina* infection at 4, 8, and 12 hpi were selected by comparing the expression levels at three time points with those 0 hpi for either Qgls8-R or Qgls8-S (4 hpi vs 0 hpi in Qgls8-R or Qgls8-S, 8 hpi vs 0 hpi in Qgls8-R or Qgls8-S, 12 hpi vs 0 hpi in Qgls8-R or Qgls8-S).

Weighted gene co-expression network analysis (WGCNA) was performed on all expressed genes across 24 samples using the R package. The analyzed genes met the following criteria: an average TPM value exceeding 1 and a ranking within the top 20,000 genes in descending order according to Mean Absolute Deviation (MAD) across 24 samples. A soft thresholding power of 22 was used to calculate the adjacency matrix and topological overlap matrix (TOM) using Pearson’s correlation. Gene modules with at least 30 genes were detected using the dynamic cutting algorithm and merged with a default cutoff value (0.25).

### 2.5 Quantitative analysis of jasmonic acid, salicylic acid and their derivatives

The *C. zeina*-inoculated fourth leaves of Qgls8-R and Qgls8-S were collected at 0 hpi and 24 hpi. For each sample, three biological replicates were set up, and each biological replicate consisted of five plants. Salicylic acid (SA), salicylic acid 2-O-β-glucoside (SAG), jasmonic acid (JA), jasmonoyl-L-isoleucine (JA-Ile), cis(+)-12-oxophytodienoic acid (OPDA), 2, 3-dihydroxybenzoic acid (2,3-DHBA), 2, 5-dihydroxybenzoic acid (2,5-DHBA), and L-phenylalanine (Phe) were extracted and analyzed in Henan University. They were quantified by ultra-high-performance liquid chromatography-tandem mass spectrometry (UPLC-MS/MS). Samples were analyzed using a Xevo TQ-XS system (Waters, USA) equipped with an ESI ion source. Data analysis was performed using the spectrometer software (Masslynx v.4.2).

### 2.6 Gene expression analysis by RT‐qPCR

Total RNAs of maize leaves were extracted using E.Z.N.A.™ Plant RNAKit (OMEGA) and converted into cDNA using FastKing RT SuperMix (Tiangen) following the manufacturer’s instructions. The cDNA of all samples was diluted six times as a template. Gene-specific primers were designed using Primer 5.0 (**Supplementary Table S14**). RT‐qPCR was performed using RealUniversal Color PreMix (Tiangen) and run on QuantStudio™ 7 Flex system. The maize *ZmEF1α* was used as internal control.

## 3 Results

### 3.1 Phenotypic evaluation of Qgls8-R and Qgls8-S for GLS resistance

**Figure 1:**
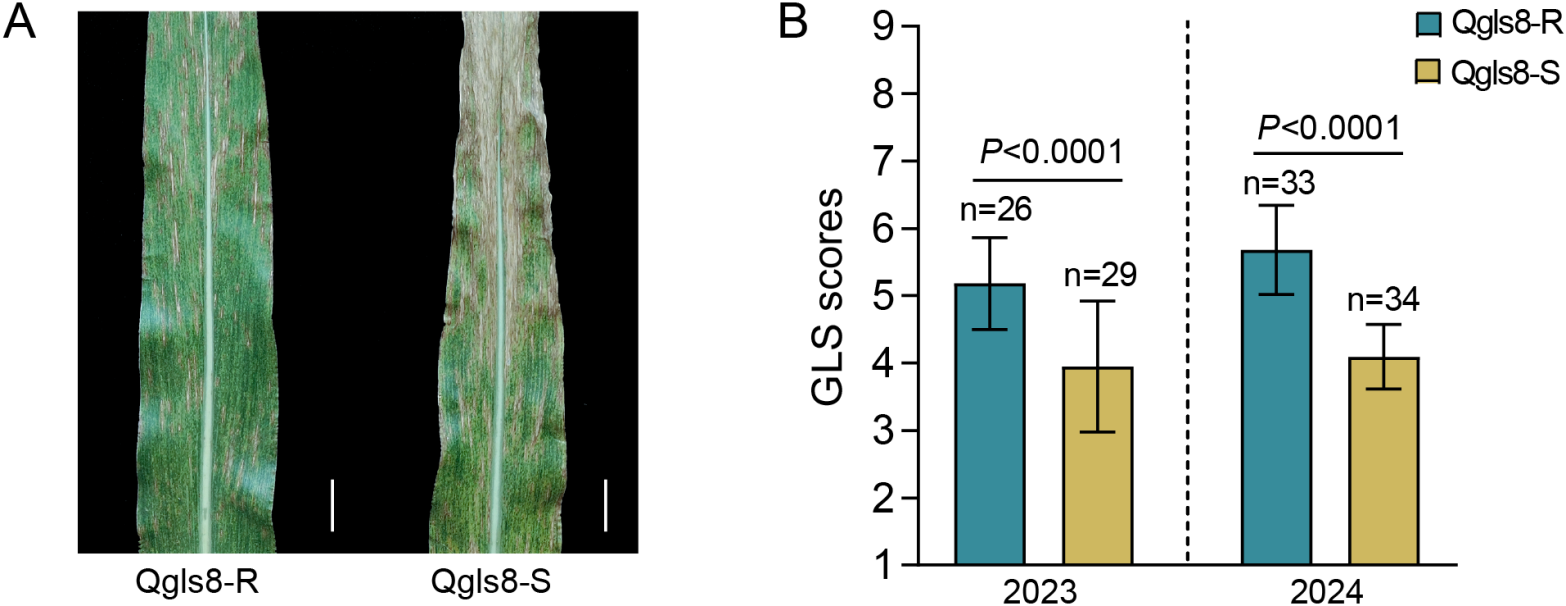
Phenotype evaluation of Qgls8-R and Qgls8-S for GLS resistance. **(A)** GLS symptom of Qgls8-R and Qgls8-S in field trials. Scale bars corresponds to 2.5 cm. **(B)** GLS scores of Qgls8-R and Qgls8-S. Disease scoring, 1–9 scales; 1 indicates the most susceptible score and 9 indicates the most resistant score. Data are presented as all the mean values ± SD. Significant differences were determined by Student’s *t*-test. The “n” indicates the number of plants for GLS assessment.

Using NIL populations in which segments of the teosinte genome had been introgressed into the maize B73 background, the QTL *Qgls8* for GLS resistance was previously mapped into a ~130-kb region in bin 8.06 (Lennon et al., 2016; Zhang et al., 2017). A pair of NILs Qgls8-R and Qgls8-S was developed from segregating populations derived from the NIL parents Z032E0081 and Z033E0056 with contrasting teosinte alleles at *Qgls8*. The allele from Z032E0081 confers increased GLS resistance, while allele from Z033E0056 confers decreased GLS resistance compared with B73 (Zhang et al., 2017). Assessment of the genetic backgrounds using the Maize6H-60K array revealed that the similarity between Qgls8-R and Qgls8-S was about 95.75% (**Supplementary Table S1**). To evaluate the GLS resistance, Qgls8-R and Qgls8-S were infected by fungal pathogens of GLS naturally in the field trials in Tongchuan, Shaanxi Province in 2023 and 2024. Based on the 1–9 scale assessment, the GLS score of Qgls8-R was significantly higher than that of Qgls8-S (**Figures 1A, B**). These results indicate that the allele from Qgls8-R confers enhanced resistance to GLS in the field.

### 3.2 Transcriptome profiling of the NILs pair in response to *C. zeina* infection

**Figure 2:**
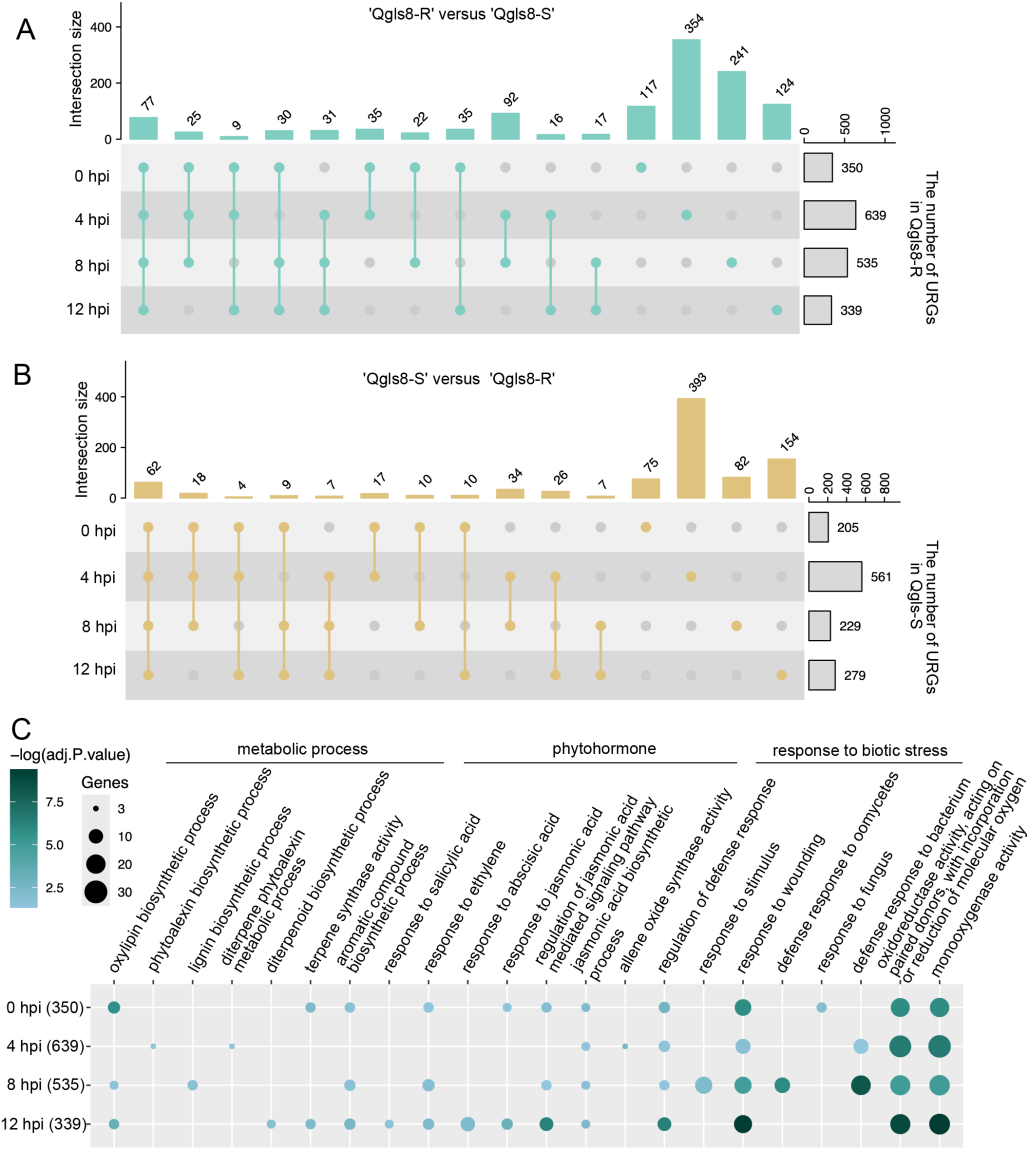
Comparison analysis of gene expression at the four time points after inoculation with *C. zeina* between Qgls8-R and Qgls8-S. **(A)** The number of up-regulated genes (URGs, |Log2FoldChange| > 0.58, padj ≤ 0.05) from ‘Qgls8-R versus Qgls8-S’ among the four comparison groups (Qgls8-R vs Qgls8-S at 0 hpi, 4 hpi, 8 hpi and 12 hpi). **(B)** The number of URGs from ‘Qgls8-S versus Qgls8-R’ among the four comparison groups (Qgls8-S vs Qgls8-R at 0 hpi, 4hpi, 8 hpi and 12 hpi). **(C)** GO analysis of the URGs from ‘Qgls8-R versus Qgls8-S’ at four time points. All the GO terms were shown with a *P*-value less than 0.05. URGs were annotated into three categories: biological processes, cellular components, and molecular functions.

A total of 24 samples were collected from Qgls8-R and Qgls8-S at 0, 4, 8, and 12 hpi for transcriptome profiling. The mapping rates of the 24 transcriptome datasets of both Qgls8-R and Qgls8-S ranged from 93.94% to 96.61% (**Supplementary Table S2**). Principal Component Analysis (PCA) revealed that the biological replicates of each sample grouped together (**Supplementary Figure S1**). Differential expression was conducted to explore the transcriptome differences between Qgls8-R and Qgls8-S. A total of 1,225 URGs were identified in Qgls8-R compared with Qgls8-S, which included 350, 639, 535, and 339 URGs at 0 hpi, 4 hpi, 8 hpi, 12 hpi, respectively. While there were 908 URGs in Qgls8-S compared with Qgls8-R, including 205, 561, 229, and 279 URGs at 0 hpi, 4 hpi, 8 hpi, and 12 hpi, respectively (**Figures 2A, B**). The highest number of URGs was found at 4 hpi in both Qgls8-R and Qgls8-S, indicating that 4 hpi might be a key time point for Qgls8-R and Qgls8-S to defend against *C. zeina* attack.

Gene Ontology (GO) analysis was performed to identify enriched GO terms for URGs in Qgls8-R and Qgls8-S, respectively. The 350 URGs at 0 hpi in Qgls8-R were enriched for defense response associated processes including response to fungus, JA biosynthesis process, and response to wounding (**Figure 2C**; **Supplementary Table S3**). Similarly, the URGs at 4, 8, and 12 hpi in Qgls8-R were related to the JA biosynthesis process and response to wounding as well (**Figure 2C**, **Supplementary Table S3**). In contrast, the 205 URGs at 0 hpi in Qgls8-S did not show significant enrichment in any GO terms. Notably, the URGs at 4, 8, and 12 hpi in Qgls8-S were mostly involved in plant growth-associated processes including photosynthesis, chloroplasts, and DNA replication (**Supplementary Table S3**). The GO enrichment analysis revealed that the URGs in Qgls8-R were mainly involved in defense response, which may contribute to its GLS resistance.

### 3.3 *C. zeina*-induced URGs displayed significantly higher expression in Qgls8-R than in Qgls8-S and were associated with defense signaling pathways

**Figure 3:**
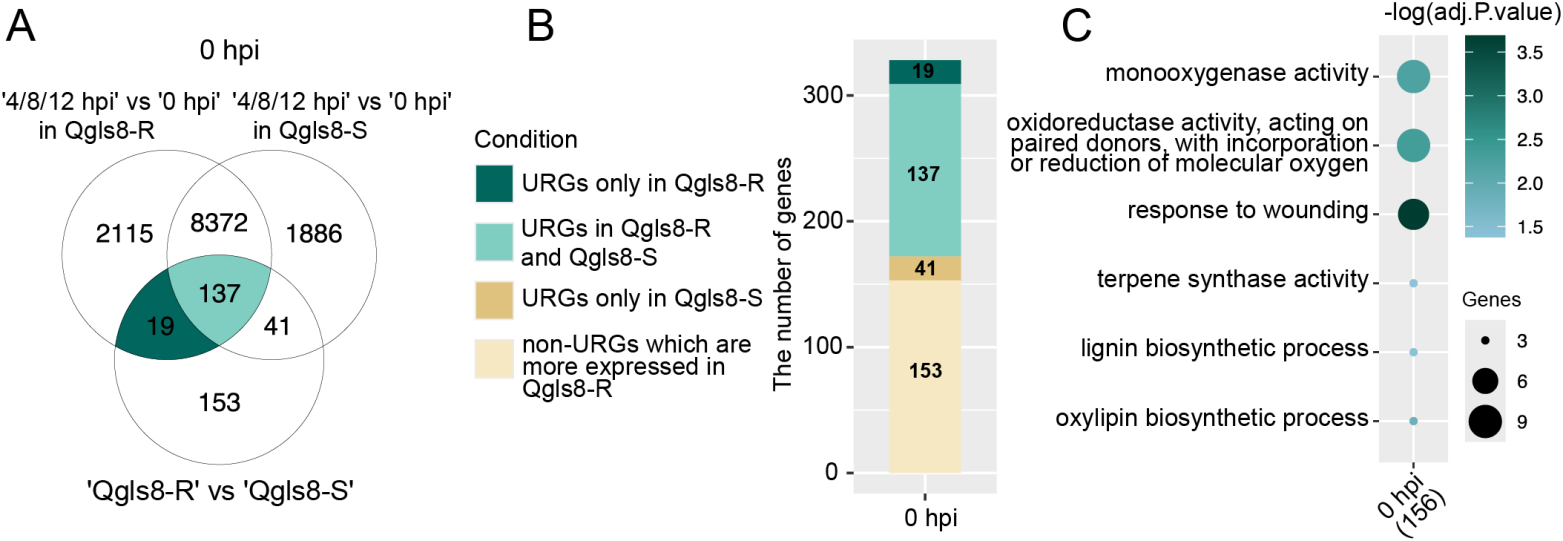
Identification of *C. zeina*-induced URGs that were more highly expressed in Qgls8-R at 0 h after *C. zeina* infection. **(A)** Upset plots displaying the number of overlapping URGs in seven comparison groups. **(B)** The number of URGs and non-URGs induced by *C. zeina* among 350 URGs identified in Qgls8-R compared with Qgls8-S at 0 hpi. **(C)** GO enrichment analysis of 156 genes that were not only up-regulated induced by *C. zeina* but also expressed at a higher level in Qgls8-R compared with Qgls8-S at 0 hpi. All the GO terms were shown with a *P*-value less than 0.05. URGs were annotated into three categories: biological processes, cellular components, and molecular functions.

A total of 350 URGs were identified in Qgls8-R compared with Qgls8-S at 0 hpi (**Figure 2A**). To further identify *C. zeina*-induced URGs, we compared the expression changes of both Qgls8-R and Qgls8-S at the three time points (4 hpi, 8 hpi, and 12 hpi) to their respective 0 hpi (4 hpi vs 0 hpi in Qgls8-R or Qgls8-S, 8 hpi vs 0 hpi in Qgls8-R or Qgls8-S, 12 hpi vs 0 hpi in Qgls8-R or Qgls8-S) (**Figure 3A**). We found 19 out of the 350 URGs were up-regulated by *C. zeina* infection exclusively in Qgls8-R and 137 URGs were induced in both Qgls8-R and Qgls8-S (**Figure 3B**; **Supplementary Table S4**). In total, 156 URGs which displayed higher expression levels in Qgls8-R and were induced by *C. zeina* were performed for GO enrichment analysis. These URGs were mostly related to monooxygenase activity, response to wounding, terpene synthase activity, lignin biosynthetic process, and oxylipin biosynthetic process (**Figure 3C**; **Supplementary Table S5**).

**Figure 4:**
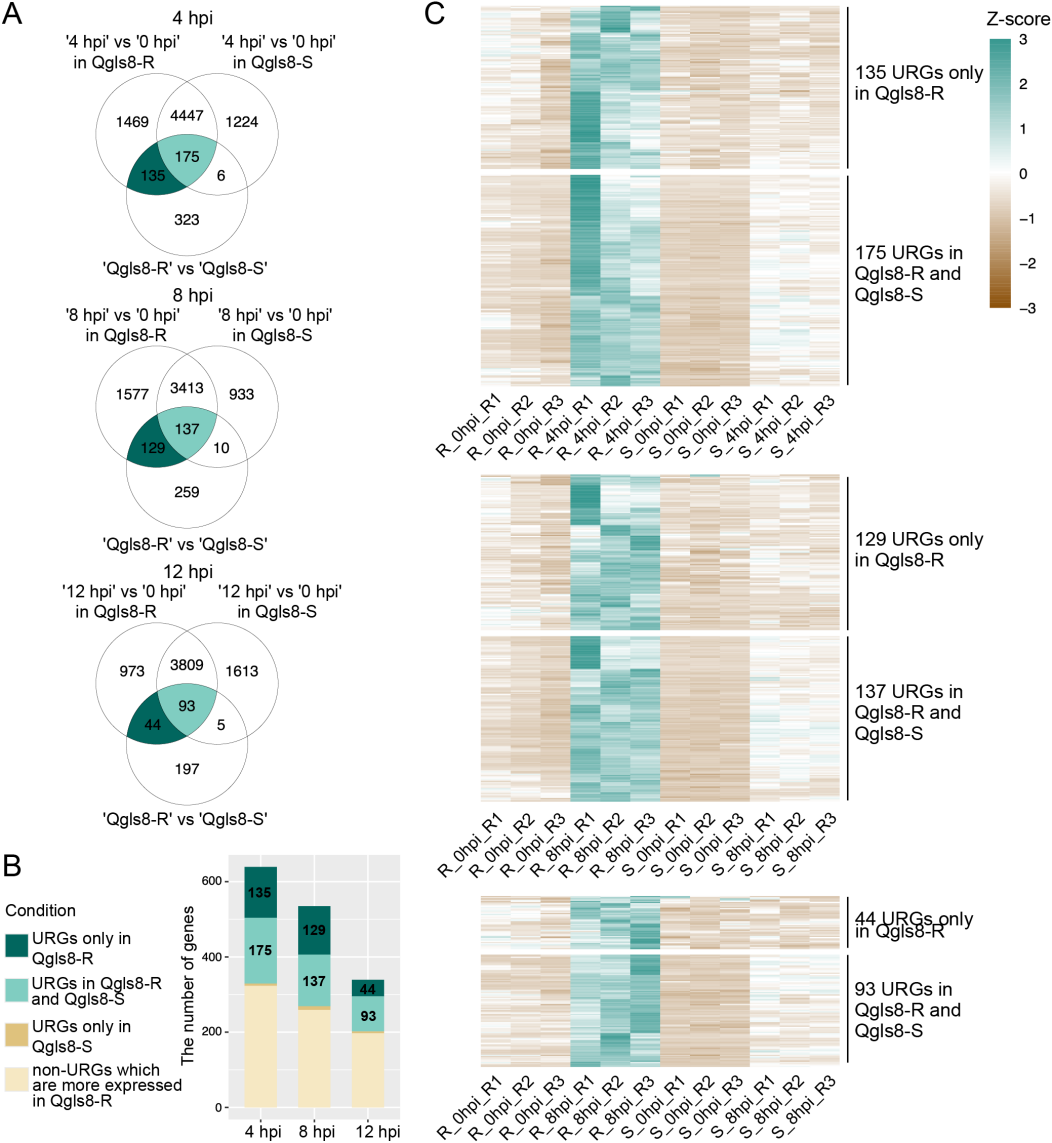
Identification of *C. zeina*-induced URGs that were expressed at a higher level in Qgls8-R compared with Qgls8-S at 4 h, 8 h, and 12 h post inoculation (hpi) with *C. zeina*. **(A)** Vene diagram showing the number of overlapping URGs in three comparison groups at 4 hpi, 8 hpi, and 12 hpi, respectively. **(B)** The number of URGs and non-URGs induced by *C. zeina* among 639, 535, and 339 URGs at 4 hpi, 8 hpi, and 12 hpi, respectively. **(C)** Expression profiles of URGs after *C. zeina* infection using transcripts per million (TPM). R, Qgls8-R; S, Qgls8-S.

**Figure 5:**
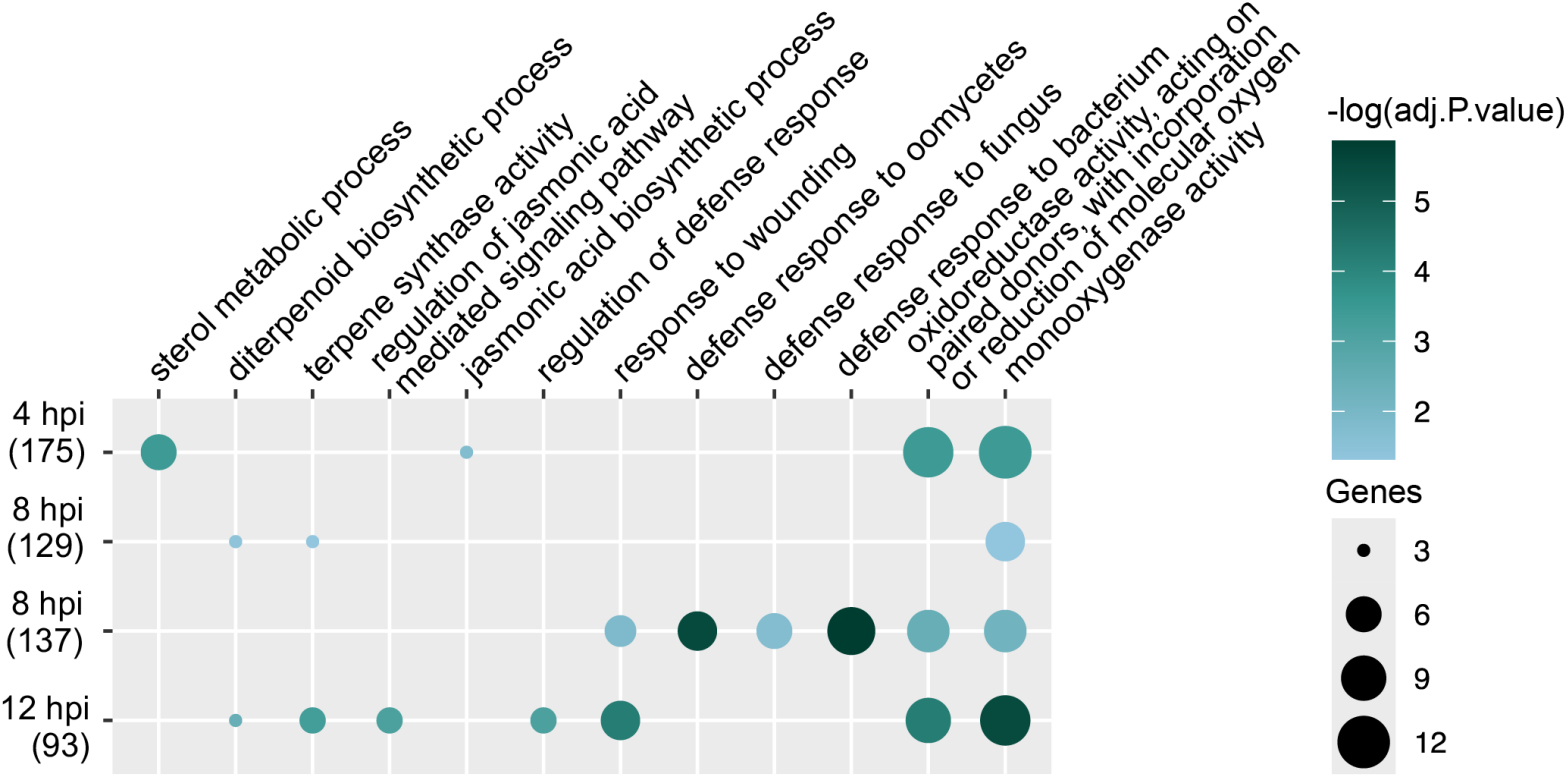
GO enrichment analysis of *C. zeina*-induced URGs at 4 hpi, 8 hpi, and 12 hpi, respectively. These genes were induced by *C. zeina* and expressed more highly in Qgls8-R compared with Qgls8-S at 4 hpi, 8 hpi, and 12 hpi, respectively. All the GO terms were shown with a *P*-value less than 0.05. URGs were annotated into three categories: biological processes, cellular components, and molecular functions.

To further analyze *C. zeina*-induced URGs, we identified genes exhibiting up-regulated response to the pathogen and higher expression after *C. zeina* inoculation in Qgls8-R compared with Qgls8-S. *C. zeina*-induced URGs at 4, 8, and 12 hpi for either Qgls8-R or Qgls8-S were selected by comparing the expression levels at three time points to 0 hpi (4 hpi vs 0 hpi in Qgls8-R or Qgls8-S, 8 hpi vs 0 hpi in Qgls8-R or Qgls8-S, 12 hpi vs 0 hpi in Qgls8-R or Qgls8-S). Furthermore, by comparing expression at the three time points between Qgls8-R and Qgls8-S (Qgls8-S 4 hpi vs Qgls8-R 4 hpi, Qgls8-S 8 hpi vs Qgls8-R 8 hpi, Qgls8-S 12 hpi vs Qgls8-R 12 hpi), the URGs displaying higher expression in Qgls8-R at 4 hpi, 8 hpi, and 12 hpi were identified. There were 135, 129, and 44 URGs exclusive to Qgls8-R at 4 hpi, 8 hpi, and 12 hpi, respectively (**Figures 4A, B**; **Supplementary Tables S6**-**S8**). A total of 175, 137, and 93 URGs were identified in Qgls8-R and Qgls8-S at 4 hpi, 8 hpi, and 12 hpi, respectively (**Figure 4A, B**; **Supplementary Tables S6**-**S8**). The expression profiling of these genes revealed that all URGs had the lowest expression at 0 hpi of Qgls8-R and Qgls8-S (**Figure 4C**; **Supplementary Tables S6**-**S8**). URGs exclusive to Qgls8-R were up-regulated at 4 hpi, 8 hpi, and 12 hpi, reaching higher expression levels in Qgls8-R after *C. zeina* infection (**Figure 4C**, **Supplementary Tables S6**-**S8**), whereas the expression of those genes remained unchanged at 4, 8, and 12 hpi in Qgls8-S after *C. zeina* infection (**Figure 4C**, **Supplementary Tables S6**-**S8**). The URGs in both Qgls8-R and Qgls8-S were up-regulated to significantly higher expression levels at 4, 8, and 12 hpi in Qgls8-R. In contrast, those genes showed slight up-regulation exclusive to Qgls8-S after *C. zeina* infection (**Figure 4C**; **Supplementary Tables S6**-**S8**). All URGs were functionally classified using GO analysis. The URGs of Qgls8-R were enriched in metabolic processes such as monooxygenase activity, oxidoreductase activity, terpene synthase activity, diterpenoid biosynthetic process, sterol metabolic process, and defense responses including against bacterium, fungus, oomycetes, wounding, regulation of defense response, JA biosynthetic process, regulation of JA mediated signaling pathway, etc. (**Figure 5**; **Supplementary Table S9**). Furthermore, we identified genes exhibiting specifically down-regulated in Qgls8-R response to *C. zeina* inoculation and higher expression in Qgls8-R compared with Qgls8-S at four time points. A few genes were found (**Supplementary Figure S2**). Given the limited number of genes in the subset, GO enrichment analysis was not carried out. These results indicate that Qgls8-R might enhance GLS resistance by upregulating genes related to terpenoid metabolism, the JA signaling pathway, and defense responses against *C. zeina* infection.

### 3.4 Terpene-related genes were activated in response to *C. zeina*

**Figure 6:**
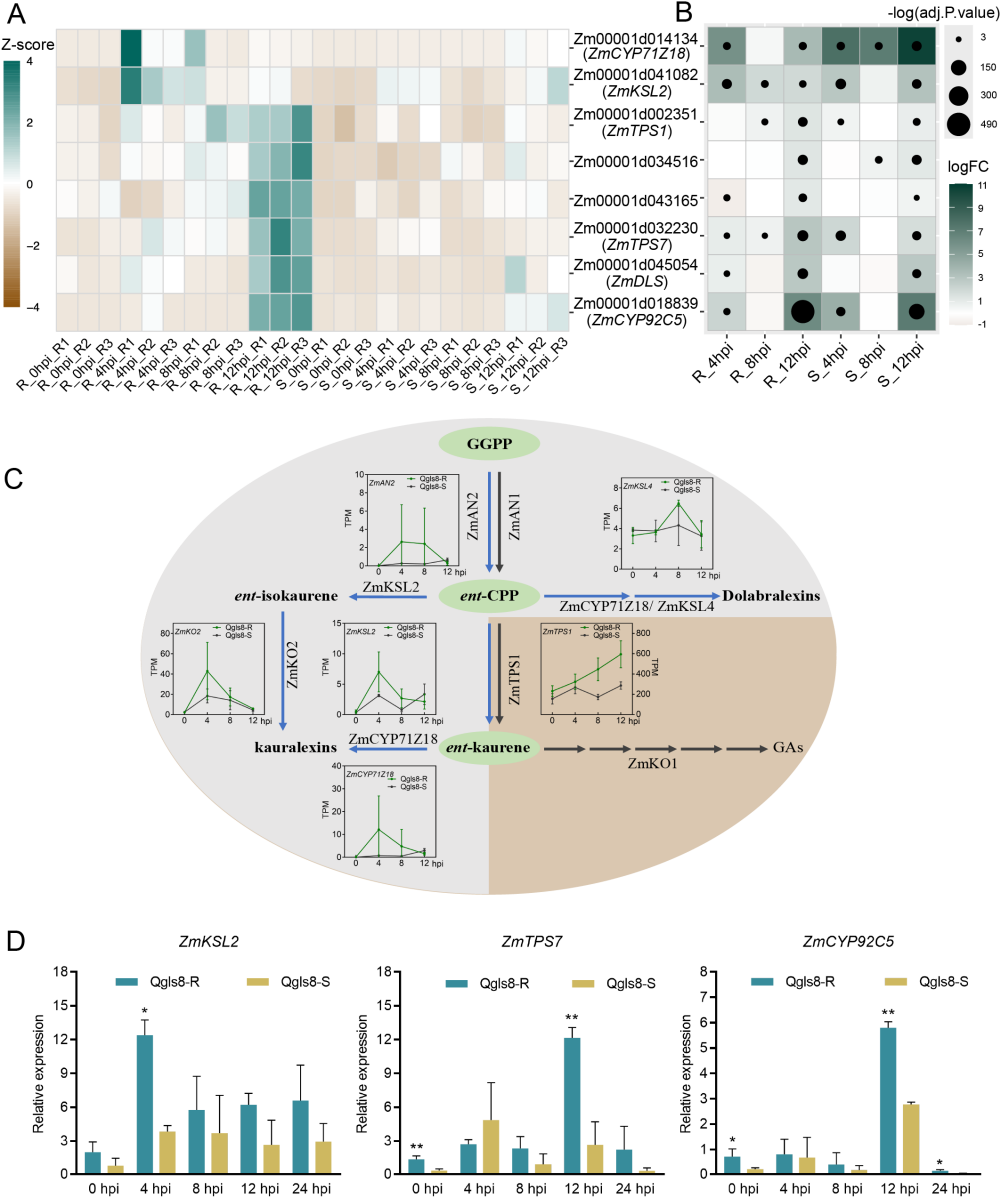
The expression of genes involved in terpenoid metabolism after *C. zeina* infection. **(A)** Expression of terpene-related genes at four time points using transcripts per million (TPM). **(B)** The expression analysis of terpene-related genes induced by *C. zeina*. Log2 fold-change of transcripts per million (TPM) at 4 hpi, 8 hpi, and 12 hpi relative to 0 hpi in Qgls8-R or Qgls8-S. The upregulated expression of genes is labeled with blue, the downregulated expression with yellow, and the unchanged expression with white, respectively. **(C)** Expression pattern analysis of genes involved in the biosynthesis of kauralexin and gibberellic acid (GA) in maize. Blue arrows, kauralexins pathways; gray arrows, GA pathways; TPM, transcripts per million. **(D)** The expression pattern analysis of *ZmKSL2*, *ZmTPS7*, and *ZmCYP92C5* after *C. zeina* inoculation by RT-qPCR. Data are presented as the mean values ± SD from three repeats. Asterisks represent significant differences by Student’s *t*-test (*0.01 < *P* < 0.05, **P < 0.01).

GO enrichment analysis revealed that eight URGs were involved in terpenoid metabolic pathway, including *ZmTPS1*, *ZmTPS7*, *ZmKSL2*, *ZmDLS*, *ZmCYP71Z18*, *ZmCYP92C5*, *Zm00001d034516*, and *Zm00001d043165*. At the four time points in the NILs, *ZmKSL2* and *ZmCYP71Z18* showed higher expression at 4 hpi in Qgls8-R. The expression of genes including *ZmTPS1*, *ZmTPS7*, *ZmDLS*, *ZmCYP92C5*, *Zm00001d034516*, and *Zm00001d043165* peaked at 12 hpi in Qgls8-R with *ZmTPS1*, *ZmTPS7*, *Zm00001d034516*, and *Zm00001d043165* displaying a gradual transcriptional increase after *C. zeina* infection. In the susceptible line Qgls8-S, these genes did not exhibit specific response patterns (**Figure 6A**). All eight terpene-related genes were significantly induced at least at one infection time point in Qgls8-R and Qgls8-S with a significant increase at 12 hpi in both lines (**Figure 6B**). Furthermore, five genes involved in diterpenoid (kauralexins) pathways were induced by *C. zeina* (**Figure 6C**). The expression of *ZmKSL2*, *ZmTPS7*, and *ZmCYP92C5* was also verified by RT‐qPCR (**Figure 6D**). These results suggest that Qgls8-R effectively responds to *C. zeina* infection through terpenoid metabolism.

### 3.5 Jasmonic acid-related genes were involved in resistance against *C. zeina*

**Figure 7:**
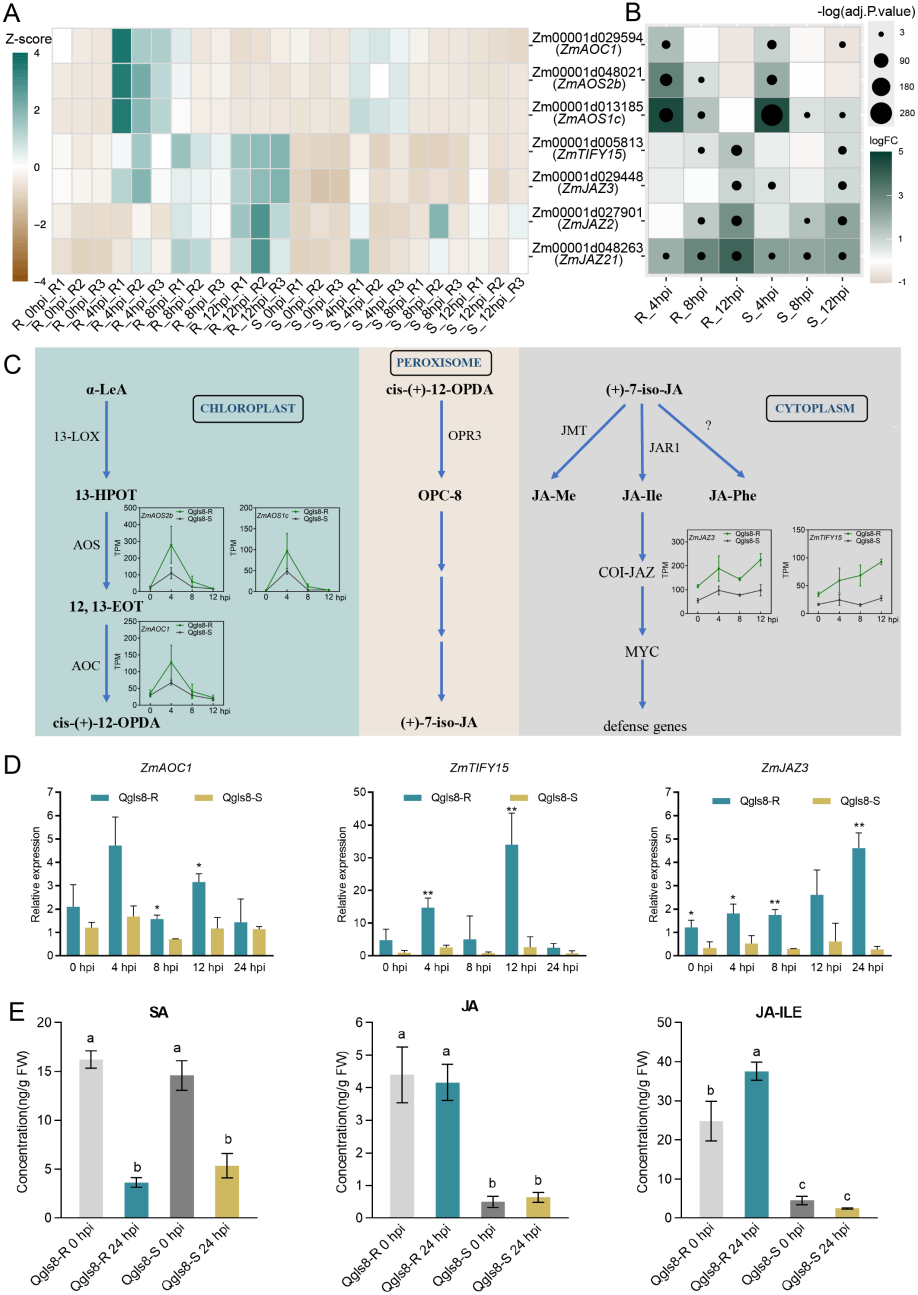
The expression of genes related to Jasmonic acid pathway after *C. zeina* infection. **(A)** Expression of Jasmonic acid-related genes at different infection time points using transcripts per million (TPM). **(B)** The expression analysis of Jasmonic acid-related genes induced by *C. zeina*. Log2 fold-change of TPM at 4 hpi, 8 hpi, and 12 hpi relative to 0 hpi in Qgls8-R or Qgls8-S. The upregulated expression of genes is labeled with blue, the downregulated expression with yellow, and the unchanged expression with white, respectively. **(C)** Expression patterns of up-regulated genes in jasmonic acid pathways. TPM, transcripts per million. **(D)** The expression pattern analysis of *ZmAOC1*, *ZmTIFY15*, and *ZmJAZ3* after *C. zeina* inoculation by RT-qPCR. Data are presented as the mean values ± SD from three repeats. Asterisks represent significant differences by Student’s *t*-test (*0.01 < *P* < 0.05, **P < 0.01). Significant differences were determined by Student’s *t*-test. **(E)** The levels of SA, JA, and JA-Ile of both Qgls8-R and Qgls8-S at 0 hpi and 24 hpi. Data are presented as all the mean values ± SD. The letters indicate a significant difference (*P*< 0.05) by one-way ANOVA with Tukey’s test.

GO analysis identified seven URGs associated with the JA signaling pathway, including *ZmAOC1*, *ZmAOS2b*, *ZmAOS1c*, *ZmTIFY15*, *ZmJAZ3*, *ZmJAZ2*, and *ZmJAZ21*. Among those, *ZmAOC1*, *ZmAOS2b* and *ZmAOS1c* displayed the highest expression levels at 4 hpi in both lines, with significantly higher levels in Qgls8-R (**Figure 7A**). Four genes showed a gradual increase in Qgls8-R, with *ZmTIFY15* and *ZmJAZ3* up-regulated from 4 hpi to 12 hpi, and *ZmJAZ2* and *ZmJAZ21* up-regulated from 8 hpi to 12 hpi (**Figure 7A**). In contrast, these four JA-related genes did not display specific expression patterns in the susceptible Qgls8-S (**Figure 7A**). Compared with 0 hpi, all seven genes were significantly up-regulated at least at one time point in both lines (**Figure 7B**). *ZmAOC1*, *ZmAOS2b*, and *ZmAOS1c* were significantly up-regulated at 4 hpi in both Qgls8-R and Qgls8-S, while *ZmTIFY15*, *ZmJAZ3*, *ZmJAZ2*, *ZmJAZ21* were most significantly induced by *C. zeina* at 12 hpi with greater up-regulation in Qgls8-R (**Figure 7B**). Five of these genes including *ZmAOS2b*, *ZmAOS1c*, *ZmAOC1*, *ZmJAZ3*, and *ZmTIFY15*, were involved in three steps of the JA signaling pathway (**Figure 7C**). The expression of *ZmAOC1*, *ZmTIFY15*, and *ZmJAZ3* was verified at 0 h, 4 h, 8 h, 12 h, and 24 h after *C. zeina* inoculation by RT-qPCR (**Figure 7D**).

To further investigate the role of the JA pathway during *C. zeina* infection, we quantified JA, SA and their derivatives at 0 h and 24 h after *C. zeina* inoculation (**Figure 7E**, **Supplementary Figure S3**). SA showed no significant difference between Qgls8-R and Qgls8-S at both time points, but decreased after *C. zeina* infection (**Figure 7E**). Notably, JA and JA-Ile in Qgls8-R were significantly higher than in Qgls8-S at 0 hpi and 24 hpi with JA-Ile showing a significant increase at 24 hpi in Qgls8-R (**Figure 7E**). These results indicate that Qgls8-R positively responds to *C. zeina* through the JA signaling pathway.

### 3.6 Analysis of a gene co-expression network in response to *C. zeina* infection

**Figure 8:**
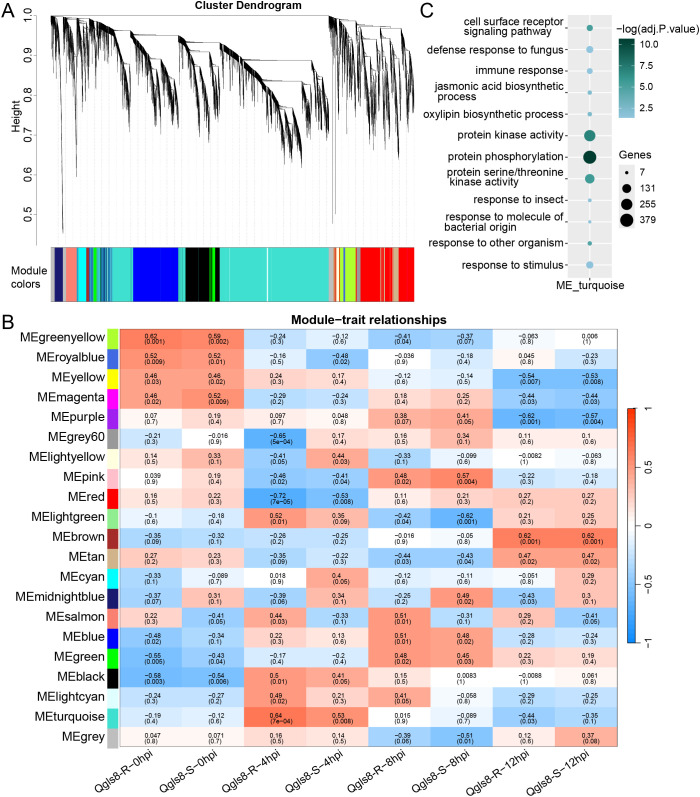
WGCNA of genes identified in both Qgls8-R and Qgls8-S at 0, 4, 8, and 12 hpi. **(A)** Hierarchical clustering tree of twenty distinct modules. Different colors are used to indicate each module. The genes that are not assigned to any module are indicated by the module “grey”. **(B)** Module-trait relationships. The module-trait correlation, ranging from -1 (blue) to 1 (red), is represented by the color scale. The corresponding correlation value is indicated on the top and the *P*-value is indicated on the bottom in each box. **(C)** GO enrichment analysis of genes identified in the module “turquoise”. All the GO terms were shown with a *P*-value less than 0.05. URGs were annotated into three categories: biological processes, cellular components, and molecular functions.

Furthermore, gene co-expression network analysis was conducted to identify genes responsive to *C. zeina* infection using weighted gene co-expression network analysis (WGCNA). Twenty distinct modules were identified, with the largest containing 5,296 genes in the module “turquoise” and the fewest 47 genes in the module “royalblue” (**Figures 8A, B**, **Supplementary Table S10**). Among these, two modules (“midnightblue” with 208 genes and “salmon” with 273 genes) displayed opposite correlations with Qgls8-R and Qgls8-S at all four time points. The module “salmon” exhibited a positive correlation with Qgls8-R and a negative correlation with Qgls8-S (**Figure 8B**), associated with diverse enzyme activities, including monooxygenase activity, oxidoreductase activity, and negative regulation of endopeptidase activity (**Supplementary Table S11**). In contrast, the module “midnightblue” was not enriched in these terms, and showed a negative correlation with Qgls8-R and a positive correlation with Qgls8-S (**Figure 8B**, **Supplementary Table S12**). Notably, the module “turquoise” had the highest positive correlation with Qgls8-R at 4 hpi (**Figure 8B**), which was related to cell surface receptor signaling pathway, protein kinase activity and phosphorylation, as well as defense response related processes, including immune response, response to fungus, and JA biosynthetic process (**Figure 8C**, **Supplementary Table S13**). The genes in the module “turquoise” were regulated at 4 hpi in Qgls8-R and Qgls8-S (**Supplementary Figure S4**). Twelve genes involved in the JA signaling pathway were identified in the module “turquoise”, including three key enzyme-coding genes Z*mAOS1c*, *ZmAOS2b*, and *ZmAOC1* (**Supplementary Table S13**). These results indicate that genes in the module “turquoise” might be an important component for the resistance of Qgls8-R against *C. zeina*.

## 4 Discussion

GLS significantly threatens maize production, which is caused by *C. zeae-maydis* and *C. zeina* with necrotrophic lifestyles. Plant resistance to those necrotrophic pathogens relies on defense related secondary metabolites including camalexin, glucosinolates, kauralexins and zealexins (Kliebenstein et al., 2005; Ahuja et al., 2012; Burow and Halkier, 2017), as well as phytohormones, such as ethylene, JA, abscisic acid, and auxins (Berrocal-Lobo et al., 2002; Adie et al., 2007; Llorente et al., 2008; Chen et al., 2021). This study analyzed the transcriptional differences between Qgls8-R and Qgls8-S at 0, 4, 8, and 12 hpi in response to *C. zeina*. URGs of the resistant line Qgls8-R were related to metabolic processes, phytohormones, and response to biotic stresses, while those in the susceptible Qgls8-S were associated with plant growth and development (**Figure 2**). Furthermore, the *C. zeina-*induced URGs in Qgls8-R were involved in defense signaling pathways and secondary metabolism, such as terpenoid biosynthetic process, defense response, and regulation of JA (**Figures 3**-**5**). The URGs in Qgls8-R involved in terpenoid metabolism and JA signaling may play crucial roles in GLS defense response.

Terpenoid production is catalyzed by terpene synthases (TPSs) and cytochrome P450s (Banerjee and Hamberger, 2018; Karunanithi and Zerbe, 2019), which regulate the biosynthesis of terpenoids and influence plant disease resistance, including ZmCYP71Z16, ZmCYP71Z18, ZmTPS6, and ZmTPS11 (Huffaker et al., 2011; van der Linde et al., 2011; Ding et al., 2019). ZmTPS1, ZmTPS7, ZmDLS, and ZmKSL2 catalyze sesquiterpenes, monoterpenes, and diterpenes, while ZmCYP71Z18 converts ent-CPP into dolabradiene and downstream dolabralexins (Mafu et al., 2018). ZmCYP92C5, a dimethylnonatriene/trimethyltetradecatetraene synthase, functions in the rate-limiting terpene production (Richter et al., 2016; Block et al., 2019). The expression of these genes was significantly induced by *C. zeina* with some showing continuously increased expression in Qgls8-R, suggesting a strong defense response activated through terpenoid metabolism upon *C. zeina* infection (**Figure 6**).

JA-dependent defenses play an important role in response to necrotrophic pathogens (Glazebrook, 2005). Key enzymes in JA biosynthesis include allene oxide synthase (AOS) and allene oxide cyclase (AOC) (Stenzel et al., 2012; Yang L. et al., 2023). JAZ proteins negatively regulate transcription factors in JA signaling (Vanholme et al., 2007; Chini et al., 2016). The degradation of JAZ repressor releases MYC transcription factors, which in turn induces the expression of JA-responsive genes (Chini et al., 2016). The expression of JA-related genes was significantly upregulated and the accumulation levels of JA and JA-Ile were higher upon *C. zeina* infection in Qgls8-R compared with Qgls8-S (**Figure 7**). This indicates that the JA signaling pathway is activated in Qgls8-R upon *C. zeina* infection, which may contribute to enhanced GLS resistance.

*Qgls8* has been fine-mapped to 130 kb, containing four predicted candidate genes based on B73_v4 reference genome, *Zea mays* subsp. *mexicana* genome (Zx-TIL18-REFERENCE-PanAnd-1.0; Zx-TIL25-REFERENCE-PanAnd-1.0), and *Zea mays* subsp. *parviglumis* genome (Zv-TIL01-REFERENCE-PanAnd-1.0; Zv-TIL11-REFERENCE-PanAnd-1.0) (Zhang et al., 2017; Stitzer et al., 2025). Among them, two genes are annotated as leucine-rich repeat receptor-like kinases (LRR-RLK), one as phosphoethanolamine N-methyltransferase, and one as ABC transporter. LRR-RLKs often act as PRRs to recognize pathogens in PTI, such as FLS2 (Gomez-Gomez and Boller, 2000), EFR (Zipfel et al., 2006), and Xa21 (Song et al., 1995; Ngou et al., 2022a). The perception of pathogen activates JA signal transduction (Chini et al., 2007; Thines et al., 2007; Fonseca et al., 2009; Sheard et al., 2010; Zhang et al., 2015). In addition, plants LRR-RLKs defend against pathogens or herbivores by regulating JA and terpenoids biosynthesis. Rice OsLRR-RLK1 functions upstream of the JA signaling pathway and regulates the biosynthesis of JA induced by chewing herbivores to initiate defense responses against herbivores (Hu et al., 2018). Maize *FI-RLPK* inhibits the growth of fungal pathogen *C. heterostrophus* by accumulating JA, kauralexins and zealexins in maize (Block et al., 2021). The phosphoethanolamine N-methyltransferase has not been reported in the JA signaling and terpenoid biosynthesis pathway. ABC transporters are shown to be involved in the transport of monoterpenoids, sesquiterpenes and diterpenes, such as VmABCG1 (Demessie et al., 2017), AaPDR3 (Fu et al., 2017), NbABCG1 and NbABCG2 (Shibata et al., 2016). The determination of the exact functional gene underling *Qgls8* will be interesting for future work to uncover the comprehensive network relating to *Qgls8-*mediated GLS resistance.

## 5 Conclusion

GLS is a devastating foliar disease of maize. This study used a pair of NILs Qgls8-R and Qgls8-S for transcriptome profiling to explore the molecular mechanisms underlying GLS resistance. We found that URGs in the resistant line Qgls8-R were key to immune signaling, which may contribute to increasing resistance against *C. zeina*. *C. zeina* infection significantly induced terpene- and JA-related genes with the highest expression in Qgls8-R. JA and JA-Ile accumulation levels were also significantly higher in Qgls8-R than in Qgls8-S. Genes related to JA biosynthetic process were also identified by WGCNA analysis. These results indicate that terpenoid metabolism and JA signaling pathway may be activated in response to *C. zeina*, enhancing GLS resistance.

## Data Availability

All raw RNA-seq data generated in this study have been deposited into SRA (sequence read archive) of NCBI, with the accession number PRJNA1222306. The data supporting the conclusions of this article are in the **Supplementary Files**.
