# Supplementary material for: Comparative transcriptomics in B73-teosinte near-isogenic maize lines reveals key defense signaling and phytoalexins in response to Cercospora zeina infection
Source: Front Plant Sci. 2025 Jun 5;16:1580016. doi: 10.3389/fpls.2025.1580016 (PMC12176847; doi:10.3389/fpls.2025.1580016)
Supplement: Supplementary file 1 [file DataSheet1.docx]

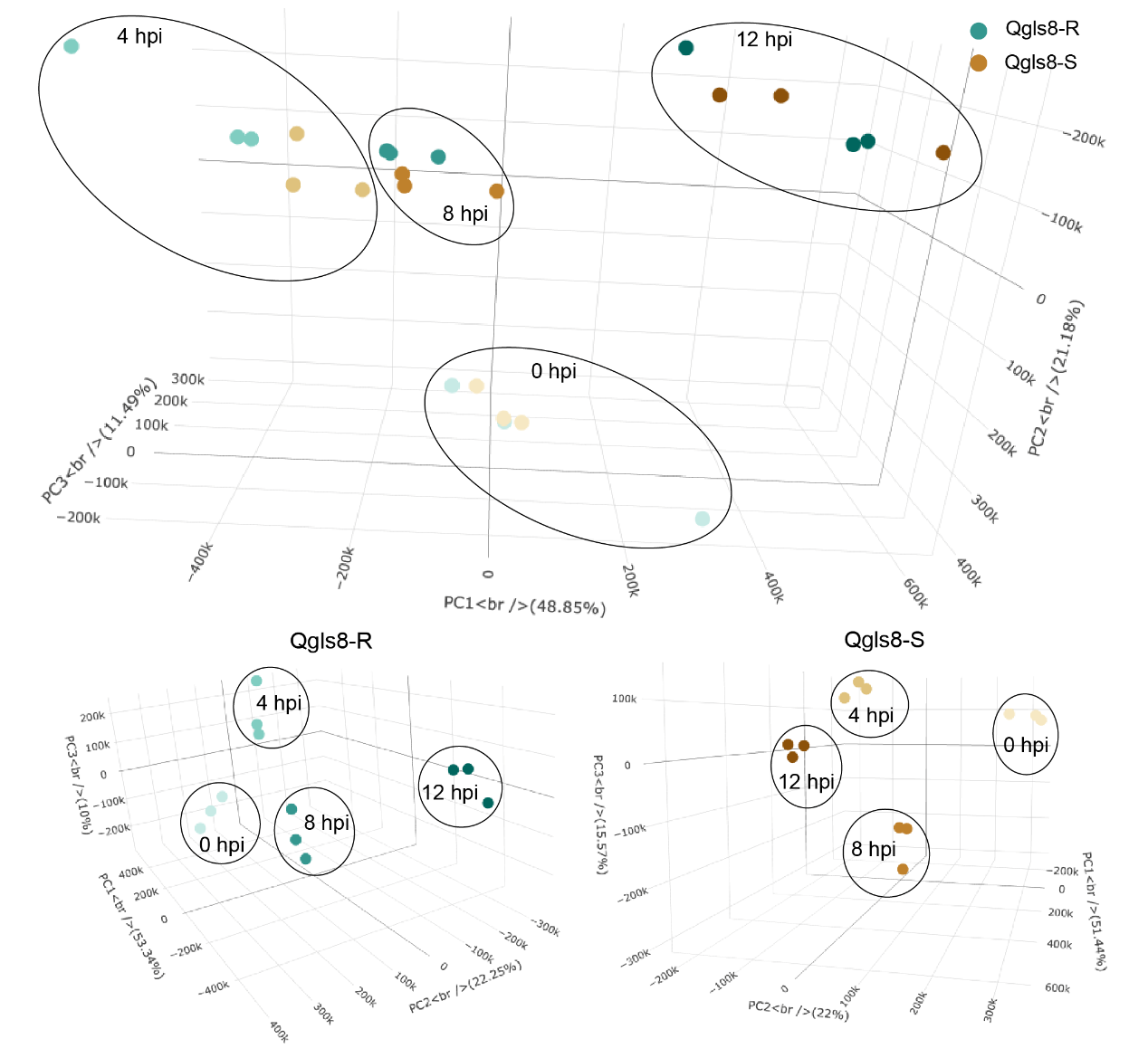


**Supplementary Figure S1** Principal Component Analysis (PCA) of gene expression. Each point represents an independent biological replicate. Points of different colors represent different samples.


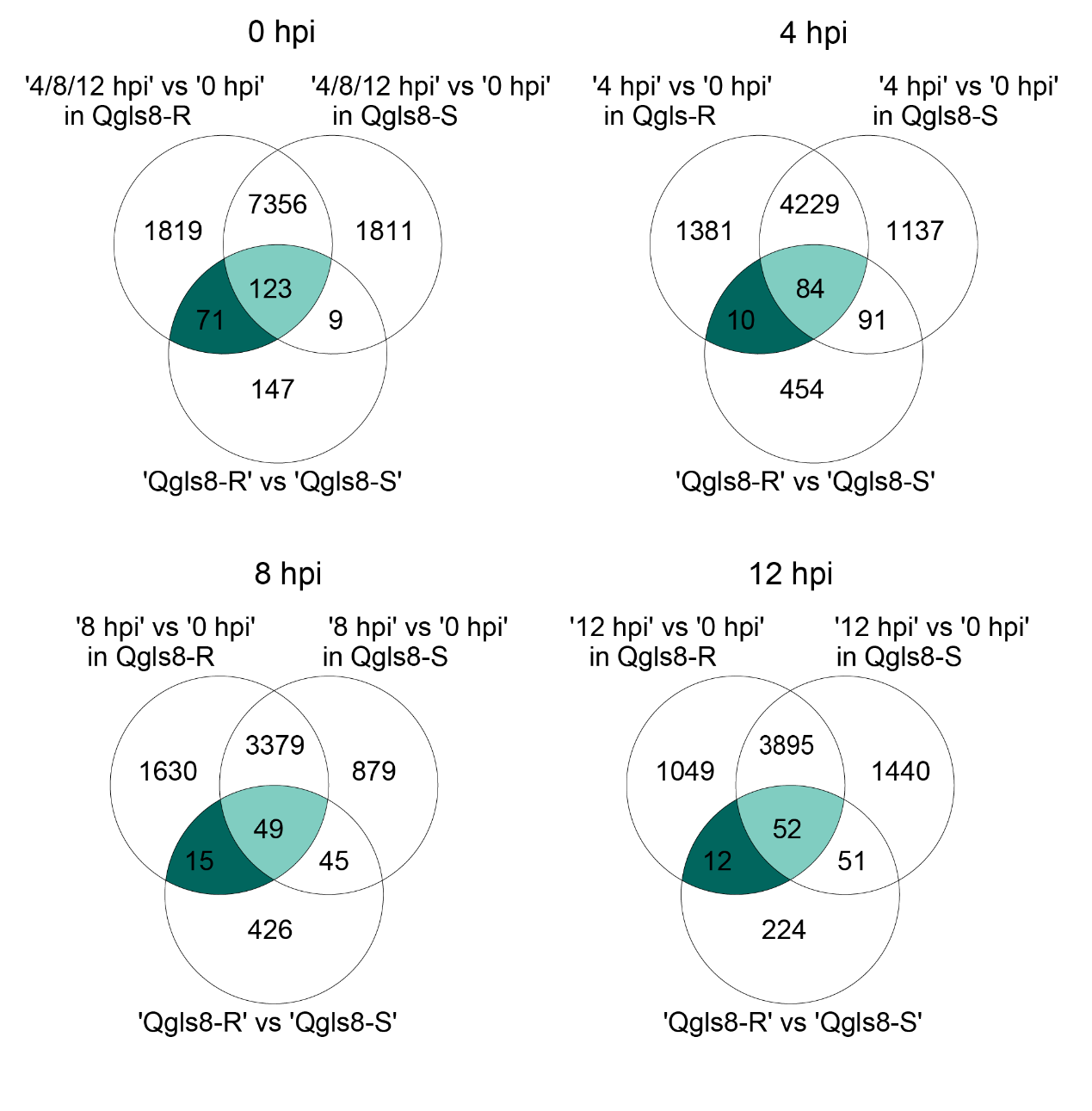


**Supplementary Figure S2** Identification of down-regulated genes induced by *C. zeina* infection that were more highly expressed in Qgls8-R. Green, down-regulated genes only in Qgls8-R; Blue, down-regulated genes in both Qgls8-R and Qgls8-S


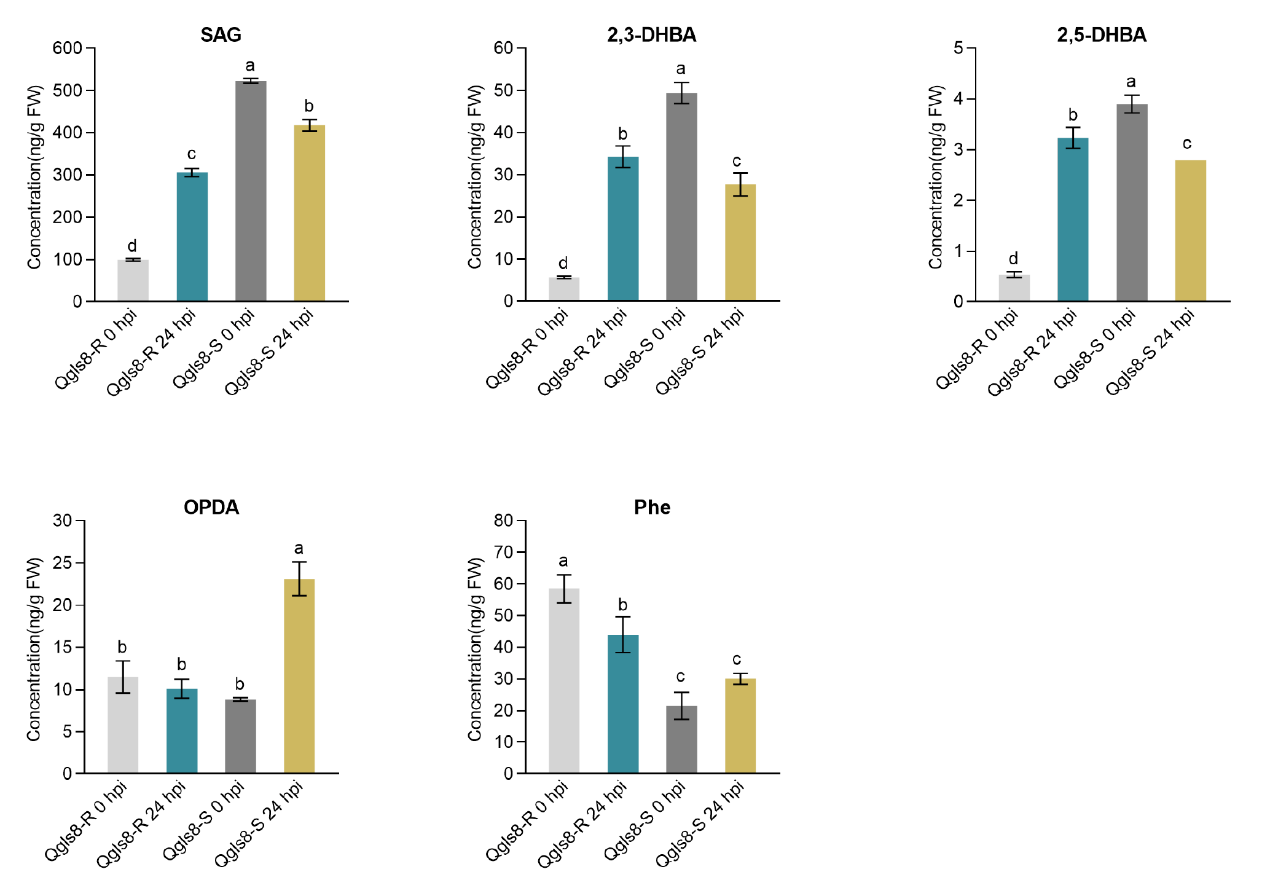


**Supplementary Figure S3** The levels of SAG, 2,3-DHBA, 2,5-DHBA, OPDA, and Phe of both Qgls8-R and Qgls8-S at 0 hpi and 24 hpi. Data are presented as all the mean values ± SD. The letters indicate a significant difference (*P*< 0.05) by one-way ANOVA with Tukey’s test. SAG, Salicylic acid 2-O-β-glucoside; 2,3-DHBA, 2, 3-Dihydroxybenzoic acid; 2,5-DHBA, 2, 5-Dihydroxybenzoic acid; OPDA, cis(+)-12-Oxophytodienoic acid; Phe, L-Phenylalanine


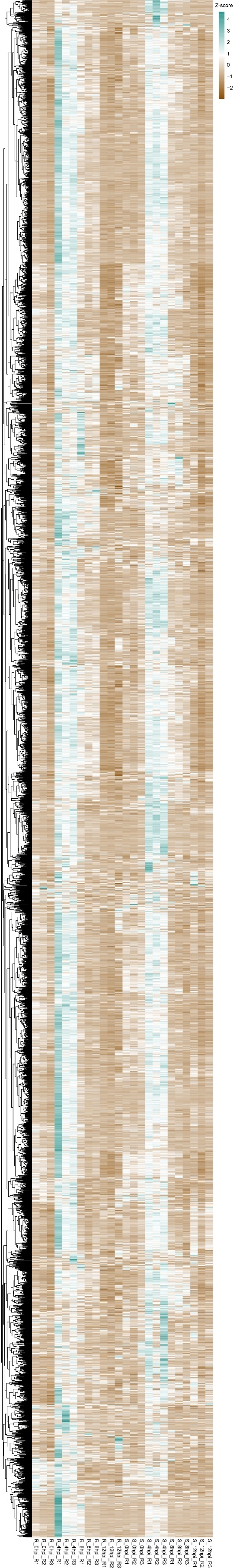


**Supplementary Figure S4** Expression profiles of all the genes in the module “turquoise” after *C. zeina* infection using transcripts per million (TPM). R, Qgls8-R; S, Qgls8-S
